# A quartet-based approach for inferring phylogenetically informative features from genomic and phenomic data

**DOI:** 10.1016/j.csbj.2025.08.015

**Published:** 2025-08-22

**Authors:** Vivian B. Brandenburg, Ben Luis Hack, Axel Mosig

**Affiliations:** a Ruhr University Bochum, Faculty of Biology and Biotechnology, Bioinformatics Group, Unversitätsstraße 150, 44801 Bochum, Germany; b Ruhr University Bochum, Center for Protein Diagnostics, Gesundheitscampus 4, 44801 Bochum, Germany

**Keywords:** Neural network, Distance based phylogeny, Phenotype evolution

## Abstract

Neural networks are widely used in bioinformatics to extract features from morphological, structural, and sequence data of different taxa. A key question is whether such features are compatible with a known phylogenetic tree describing the evolutionary relationships among the taxa. We address this question with a machine learning approach that takes taxon-specific data and a reference tree as input, and trains a neural network to produce a latent feature space whose pairwise distances are consistent with the tree topology. Our approach builds on the established role of quartets in distance-based phylogeny, leading to a quartet-based loss function for neural network training. In a proof-of-concept study using bacterial ribosomal RNA sequences, we show that the learned feature distances closely match the reference phylogeny. This framework can be applied to diverse biological data types, providing a principled way to incorporate phylogenetic constraints into neural network–based feature extraction.

## Graphical abstract

**Figure:**
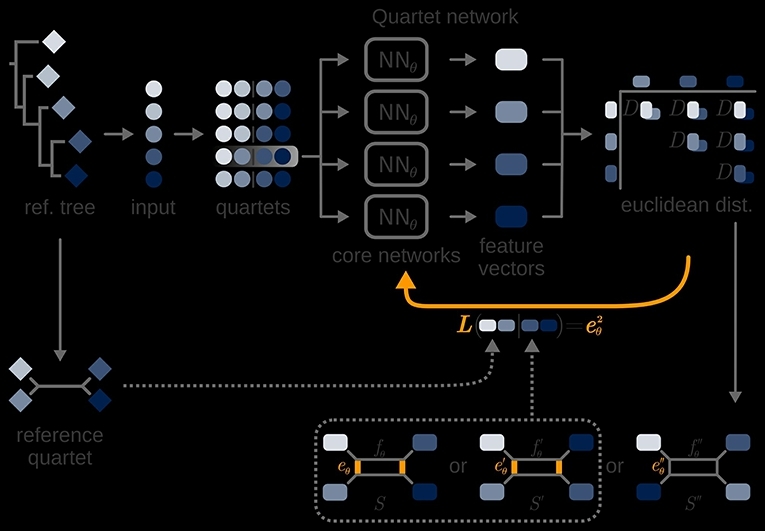

## 1 Introduction

Using morphological or other phenotypic information to investigate the evolution of organisms is of high relevance in many settings. Consequently, involving phylogenetic information into structure prediction is a commonly approved practice in the prediction of RNA secondary structure [16] as well as protein structure [21]. Since structural or more generally, phenotypic features tend to be more difficult to capture than genomic characters, many recent studies employ deep neural networks to extract numeric features from phenotype data [15], [12], [2], [18]. As Adaïmé et al. [2] have pointed out, such approaches lead to a natural and highly important question: How can neural networks be trained to yield feature representations that can be considered phylogenetically plausible? Our present methodological contribution addresses this important question by contributing a deep learning approach that builds on the mathematical structure underlying distances among quartets [3] as a well-established foundation of distance-based phylogeny.

Our work follows the perspective taken by Adaïmé et al. [2] as well as other authors [44] that neural networks can be considered feature extractors in a natural manner. Multi-layered neural networks are commonly trained for biological classification of images or other phenotype data into *k* different taxa [45] by passing the input data into the first layer of the network and processing them through hidden layers into the final output layer, which yields the classification. The neurons in the hidden layers of the network induce abstract representations of the input data [4], which are usually captured in the last non-output layer of the multi-layered neural network. However, as has been pointed out by Adaïmé et al. [2], these features bear little phylogenetic information in a purely classifying neural network, as they have been inferred to optimize the distinction between classes without any evolutionary presupposition.

The lack of evolutionary presuppositions in neural network derived features impedes their use in phylogenetic analyses, which has been noted in several recent studies. For example, Davis et al. [12] note that the performance of neural networks trained to count reproductive structures in digitized herbarium specimens is inconsistent with phylogenetic relatedness, and thus identify a need to further investigate the relationship between phylogeny, morphological diversity and neural network model transferability. A similar mismatch between a neural network latent space and phylogeny is observed in a variational autoencoder trained to reconstruct primate mandibles by Tsutsumi et al. [44]. Adaïmé et al. [2] investigated the use of neural networks for the phylogenetic placement of pollen images of extinct taxa. As the authors point out, using features that do not represent, or at least approximate, phylogenetic characters make it impossible to use them for the intended phylogenetic placement. In [18], the authors use neural network derived features to test for signals of mimicry groups in subspecies of *Heliconius erato* and *Heliconius melpomene*, where neural network derived features are used to unveil phenotypic effects mimicry. In more general terms, the recent review by He et al. [15] points out the potential ability of neural networks to compensate distortions often inherent to fossils that lead to distorted morphometrics. In short, recent studies that utilize neural networks for morphometric feature extraction underline the importance of phylogenetic considerations when training such networks.

The relevance of inferring phylogenetically informative features extends beyond morphological data to lower-level phenotypes, in particular in structural biology. This is well underlined by the recent success in protein structure prediction by the AlphaFold 2 system [21], which takes multiple sequence alignments as input and explicitly translates evolutionary information into spatial relationships through the so-called evoformer blocks, which are the core constituents of the neural network.

In summary, approaches that identify base pairings or other patterns of interaction as phylogenetically informative entities promise potential not only for improved structure predictions but also for improved understanding of such mechanism.

### 1.1 Approach

The examples from structural biology and morphometrics introduced in the previous section illustrate an inherent problem of identifying phylogenetically informative characters: They may become apparent only after substantial processing of the data they are obtained from. Ideally, such processing steps are rooted in well-understood models for the underlying mechanisms. For example, inference of RNA base pairing patterns is based on commonly accepted energy models [25] with underlying combinatorial structures and prediction algorithms [24], [17]. Yet, for many modalities of phenotypic data other than RNA secondary structure, such a mechanistic understanding will be limited, so that fully model-based preprocessing is not an option. In fact, obtaining better insights into the mechanisms underlying a specific phenotype is a major motivation to infer phylogenetically informative characters, as we propagate in this manuscript.

**Fig. 1:**
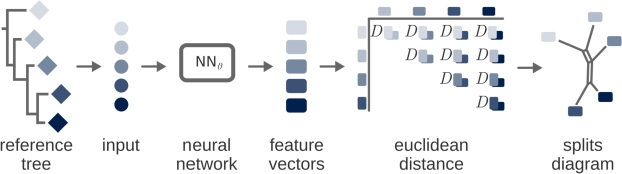
Distance-based approach to infer phylogenetically informative features. We assume input data for a given genotype or phenotype for a given set of taxa with a well-established reference tree. The data can be passed through a neural network, whose latent space establishes a vector representation of the genotype or phenotype under investigation, which in turn yields a distance matrix between the observed taxa. The neural network is then trained towards the distance matrix representing the reference tree.

Whenever a certain observed phenotype lacks an underlying explicit model or mechanism, the question arises of how to uncover features that characterize and describe the phenotype. In these cases, a neural network could serve as a source of abstract features from observations. If a neural network is trained to identify a set of taxa based on phenotypic data, the following setting is obtained: the observed data are provided as input in the first layer of the network, then the input signal is transformed layer by layer, until the final output layer provides classification into different taxa. In this setting, the neurons in the layer preceding the last layer may represent an abstract phenotypic latent space. Now, if we further suppose that the phenotype features are phylogenetically informative, then the phylogeny of the taxa under consideration should be reconstructible from this latent space. This indeed constitutes the core idea of the approach that we introduce, as illustrated in Fig. 1: we train a neural network on phenotypic data such that the phylogeny can be reconstructed from the latent space representation of the taxa under consideration.

When using neural networks to infer phylogenetically informative features, it is straightforward to measure distances between the inferred features. For a given set of taxa, this yields a distance matrix, which can be utilized to reconstruct evolutionary relationships based on well-established theory [34]. In particular, quartet-based methods [3], [8] provide an elaborate mathematical framework that plays an important role in our present work. As illustrated in Fig. 3, quartets are formed by considering combinations of four taxa, and it is well understood that quartets are the natural mathematical basis for measuring the tree-likeness of a given distance matrix [3]. In our work, we combine this trait of quartets with neural networks. The core idea is that the neural network is trained to infer a feature space that maximizes the tree-likeness of its inferred latent space.

Our approach aims to infer a latent space from genomic or phenotypic data that evolves along a given phylogenetic tree. Specifically, our first approach utilizes a Siamese neural network [7] to learn feature representations of species by minimizing the difference between pairwise Euclidean distances of feature vectors and patristic distances from the reference tree, effectively capturing phylogenetic relationships. The second approach adopts this idea with a quartet network, which evaluates the consistency of pairwise distances with the correct quartet splits, ensuring that the relationships between four species at a time align with the reference tree structure. Our methodological main contribution is the introduction of a loss function that is based on quartets of taxa, and builds on the well-understood role of quartets in distance-based phylogeny. We provide a proof-of-concept for the approach by demonstrating that a neural network can learn features from bacterial rRNA sequences that evolve along a known phylogeny of the underlying bacterial species.

### 1.2 Related work

Neural networks have gained relevance in both structural and evolutionary biology in recent years, as surveyed by Mo et al. [26]. A main motivation for their introduction was the shortcoming of evolution rate models [23], such as the general time reversible model [43], to appropriately model rate heterogeneity across sites and lineages within a given set of taxa. This weakness of evolutionary models has been extensively studied in the literature [20], [28], and has been addressed, yet only with partial success, by introducing more parameter-rich statistical models [39], [11]. In this context, a recent approach by Zou et al. [47] introduced a quartet-based approach that trains neural networks using a cross-entropy loss function and assembles the resulting quartets into a tree using quartet puzzling [40].

Several deep learning approaches in phylogeny can be found to operate on the basis of pairs or quartets of taxa, although in a substantially different manner and context than our approach. For example, the Fusang approach for phylogenetic tree inference by Wang et al. [46] uses a convolutional neural network in combination with a recurrent neural network that assess all quartets of taxa in order to accelerate quartet puzzling. In a similar direction, Suvorov et al. [41] proposed a deep convolutional neural network (CNN) to infer quartet topologies from multiple sequence alignments. Kulikov et al. [22] follow a related approach by training neural networks to predict an optimal evolution rate models along with an optimal tree topology for four taxon alignments of nucleotide or amino acid sequences. Nesterenko et al. [29] follow a different approach of machine-learning based tree inference through a transformer-based network that operates on all pairs of taxa in order to infer a distance matrix that facilitates tree inference. Beside tree inference, neural networks have also been investigated to identify and assess patterns of concordance and discordance between gene trees and species trees, such as the approach by Rosenzweig et al. [33] which also builds on considering all quartets of taxa.

Some concepts related to our work can be found in the literature on deep neural networks beyond the work related to phylogenetics. In particular, Siamese neural networks [7], contrastive loss [9] and triplet loss [35], [14] as well as quartets [22] are concepts suitable to equip neural networks with a bias towards learning hierarchical structures or distances, although this seems to be not systematically exploited in the context phylogeny, and not fully exploiting the mathematical structure behind quartets.

## 2 Methods

We presuppose a given set of *n* taxa with phenotypic observations $x_{1}\ldots ,x_{n}\in D$ in some data domain D, as well as a given unrooted (binary) phylogenetic tree *T* with the *n* taxa as leaves. We assume that this tree *T* is a well-established reference tree for the set of taxa under consideration.

Each edge *q* of the reference tree *T* partitions the *n* taxa into two groups $L_{q}$ and $R_{q}$. Correspondingly, *q* induces a set of *splits* as

$$\sigma_{q}=\{ij|k\ell :i,j\in L_{q},k,\ell \in R_{q}\}.$$

We denote the set of all quartets induced by *T* as $\Sigma_{T}:=\cup_{q}S_{q}$. Furthermore, we assume that a neural network with fixed topology is provided, whose trainable parameters are denoted by *θ*, so that the neural network computes a function

$$h_{\theta }:D\to R^{s},$$

where *s* is the dimensionality of the feature representation of the neural network. Between any pair of taxa *i* and *j*, we can now compute the distance between their feature representations as

$$D_{\theta }(i,j)=\|h_{\theta }(x_{i})-h_{\theta }(x_{j})\|.$$

**Fig. 2:**
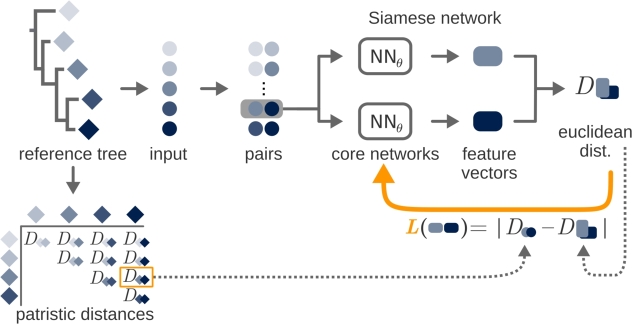
Siamese loss for training against a given reference tree. From a reference tree with five species (diamonds), input data (circles) are extracted and provided to a neural network as pairs. The model generates feature vectors (squares) for each input, and the pairwise Euclidean distance between these vectors is calculated. The siamese loss is defined as the absolute difference between this Euclidean distance and the patristic distance (summed edge length) between the corresponding species pair, and is used to optimize the model.

The resulting distance matrix $D_{\theta }$ changes with varying neural network parameters *θ*. If our aim for the neural network is to identify phylogenetically informative features, then we can phrase our goal to optimize the parameters *θ* such that the distance matrix $D_{\theta }$ obtained from $h_{\theta }(x_{1}),\ldots ,h_{\theta }(x_{n})$ complies with the established reference tree *T*. As we propose in the following, this goal can be translated into different loss functions on which the neural network can be trained, see Fig. 2.

### 2.1 Implementation

Given an input data point, the neural network function $h_{\theta }$ maps this data point to an *s*-dimensional output vector, so that we can compute all pairwise Euclidean distances between any pair of our *n* taxa, yielding the distance matrix $D_{\theta }$. In case the reference tree is not only given as a topology, but as a tree including edge lengths, we can match $D_{\theta }$ with the patristic distance induced by *T*, which we denote by $D_{T}$. This leads to the loss function

$$L_{\text{Siamese}}(\theta )=\left|D_{\theta }-D_{T}\right|,$$

which, as the name suggests, can be minimized by applying the backpropagation algorithm to a Siamese neural network topology, following the idea originally proposed by Bromley et al. [7].

Although the siamese loss function may appear intuitive at first, some of its properties are potentially dissatisfying from an evolutionary perspective. Even if we assume that the features inferred by the neural network well reflect phenotypic phylogenetic characters, it is generally unlikely that these phenotypic characters evolve time-proportional to the edge lengths in the reference tree *T*, regardless of whether the edge lengths themselves are time-proportional or not. We suggest that this problem can be avoided by defining a loss function based on quartets rather than pairs.

The quartet loss function is based on splits diagrams as the central structure that emerged from Bandelt and Dress [3]. For a subset of four taxa i,j,k,ℓ in a given distance matrix $D_{\theta }$, the splits diagram unveils how tree-like the constellation of these four taxa is in terms of $D_{\theta }$: If the four taxa are in a perfect tree-like constellation, the parallelogram in the splits diagram degenerates to a line. The important observation is that the splits diagrams obtained from $D_{\theta }$ change along with the neural network parameters *θ*. This allows us to utilize the essential properties of quartets to optimize *θ* towards supporting perfect trees that are compatible with the reference tree *T* for all quartets.

**Fig. 3:**
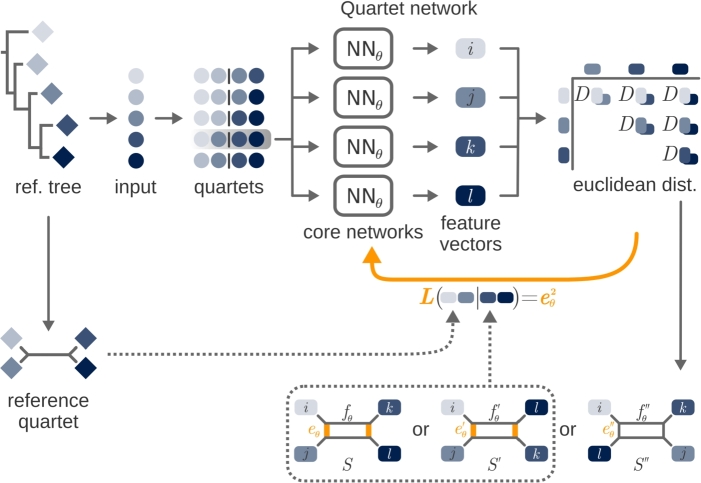
Illustration of quartet loss for training against a given reference tree. Starting from species on a reference tree (diamonds), input data (circles) are extracted and grouped into quartets. The quartets are ordered according to the structure of the reference tree. All data in a quartet are provided to a neural network, generating feature vectors (squares) for each species. The pairwise Euclidean distances between the four feature vectors are calculated and one of the three possible splits diagrams, *S*, *S*′, or *S*″, is formed from this. Compared to the reference quartet, the splits diagrams *S* and *S*′ have edges *e* and *e*′ that should be minimized to resemble the reference. Since *e*′ = −*e*″, the squared edge difference (*e*^2^) is used as the quartet loss function to optimize the model, while the splits diagram *S*″ cannot be transferred to the correct quartet.

The quartet loss is best explained by the splits diagram displayed in Fig. 3, which indicates that the edge lengths of the parallelogram $e_{\theta }$ and $f_{\theta }$ depend on the network parameters *θ*. The goal is to choose the parameters of the neural network *θ* such that the splits diagram of each quartet ij|kℓ will match the reference tree *T* as closely as possible. For a single quartet, this can be achieved through the loss term

$$L_{\text{Quartet}}^{S}(\theta )=e_{\theta }^{2},$$

where $e_{\theta }$ indicates the edge length in splits *S* to be minimized in accordance with the reference tree. The complete quartet loss function then minimizes the sum of the loss terms across all quartets $S\in \Sigma_{T}$.

The loss terms and hence the loss functions can be implemented efficiently in the closed form that yields the edge lengths

$$e_{\theta }=\frac{1}{2}(D_{\theta }(i,l)+D_{\theta }(j,k)-D_{\theta }(i,k)-D_{\theta }(j,l)).$$

*Quartet loss with Siamese regularization*  The quartet loss is minimal when the distances between species in the quartet match the topology of the reference tree. While the siamese loss is restricted to time-proportional edge lengths, the quartet loss should be considered as under-constrained, as it identifies a large set of trees with highly implausible edge lengths as equally plausible as long as they perfectly reflect the topology of the reference tree *T*. Thus, for training neural networks which infer features with plausible phylogenetic characters, it is reasonable to regularize the quartet loss by a siamese loss term weighted by a factor of *σ* as

$$L_{\text{Q+S}}(\theta )=L_{\text{Quartet}(\theta )}^{S}+\sigma \cdot \sum_{S\in \Sigma_{T}}L_{\text{Siamese}}^{S}(\theta ).$$

*Quartet scores*  While the quartet loss and siamese loss inherently serve the purpose of making the inferred distances as tree-like as possible towards the reference tree, they do not provide an absolute scale to determine how far the current loss is from an ideal representation of *T*. In fact, there is no theoretical guarantee that backpropagation will draw all quartets into the correct among the three possible splits diagrams with respect to the reference tree, as the loss landscape between different split diagrams of the same quartet can be considered discontinuous. In theory, there is no guarantee that the learning process based on the quartet loss function remains stuck in wrong splits diagrams in the case of low learning rates, or jumps randomly between the three splits diagrams if the learning rate is high. In the lack of a theoretical guarantee, we introduce the quartet score as an additional measure to how many quartets are currently in the correct splits diagram. It evaluates how well the distance matrix, induced by the current network function $h_{\theta }$, represents the reference tree *T*.

For any quartet $ij|k\ell \in S_{T}$, we determine whether the distances obtained from $h_{\theta }$ correspond to one of the splits diagrams *S*, $S^{\prime }$, or $S^{\prime \prime }$, as illustrated in Fig. 3. This is done by calculating *e* and *f* and checking their non-negativity. In cases where the distances yield splits diagrams *S* or $S^{\prime }$, it is possible to reconstruct the original split ij|kℓ by minimizing edge *e* or $e^{\prime }$. However, if the distance matrix induces the incorrect splits diagram $S^{\prime \prime }$, neither $e^{\prime \prime }$ nor $f^{\prime \prime }$ can be minimized to recover the original split.

The quartet score is defined as the fraction of quartets in the dataset that successfully reconstruct the splits *S* or $S^{\prime }$, and satisfy the condition that e<f or $e^{\prime }<f^{\prime }$. The quartet score is closely related to the quartet distance [38], which is defined as a distance between two trees. Just as related distances between trees such as Robinson-Foulds distances [5], this does not match our situation where we need to measure how well a distance matrix represents a given tree. Our quartet score measures the compatibility between a distance matrix and a reference tree in a similar, yet different manner as the quartet distance. We use the term score rather than the term distance in order to avoid confusion with the well-established quartet distance.

*Neural network topology*  We utilized a convolutional neural network (CNN) architecture for feature extraction. We essentially followed widely used standards of using CNNs for biological sequence analysis [27], [6], with a particular focus of choosing an architecture that facilitates output interpretation [30], in particular through the well-established DeepLIFT approach [36]. Input is presented to the network through one-hot encoding of the nucleic acid sequence, followed by one convolutional layer (kernel size of 10, stride of 1, 30 filters) and a subsequent average pooling layer (pool size of 5), followed by a dropout layer (20% dropout). The pooling layer was presented to two further fully connected layers with a ReLu transfer function with 360 nodes, yielding the final feature representation of 30 features.

### 2.2 Training data

*rRNA data*  In this proof-of-concept study, the first 350 nt of 16S rRNA sequences from 6 *α*-Proteobacteria and 7 *γ*-Proteobacteria were used as input data. The 16S sequences, as well as the reference tree, were downloaded from the SILVA database [31]. Each of the 16S rRNAs used was searched for the V1-V2 primer sequences from 1, which include the variable regions V1 and V2. To have an equal length for all input sequences, each 16S-rRNA was trimmed to the first 350 nt, including the reverse primer of the V1-V2 primer pair in all sequences.

**Fig. 4:**
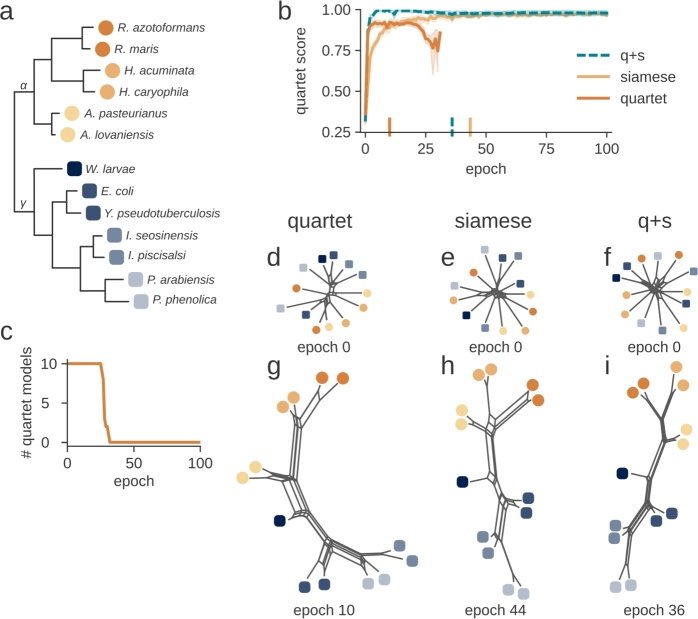
Comparison of quartet loss, siamese loss, and quartet+siamese loss for training on 16s rRNA on the reference tree. The training target is a given reference tree (a), on which 10 models are trained with quartet loss, siamese loss, and q+s loss for 100 epochs (b). During the training, the models trained with quartet loss collapse (c). For each loss function, two NeighborNet constructions are shown: Epochs 0 (d) and 10 (g) of a model trained with quartet loss, epochs 0 (e) and 44 (h) of a model trained with siamese loss, and epochs 0 (f) and 36 (i) of a model trained with q+s loss.

In order to reassure that the selected sequences contain phylogenetic signal, we reconstructed a phylogenetic tree using conventional sequence analysis. Specifically, the sequences were aligned using Clustal Omega [37], and the resulting multiple sequence alignment was used to reconstruct phylogenetic trees using Neighbor Joining [34] as implemented in the phylip software package [32]. The resulting tree shown in Fig. 4 was used as reference tree *T*.

We constructed all splits based on the reference tree *T*, obtaining $|S_{T}|=715$

*Simulated data*  To explore the use of explanatory methods, we created sequences with a stable mutation rate, without insertion or deletion mutations. For these artificial sequences, we used the reference tree from the rRNA data to guide a simulation of sequence evolution. The simulated sequences have three distinct segments:

• Segment 1: a ‘stable’ part of 8 nt, identical for all simulated sequences
• Segment 2: an ‘evolving’ part of 20 nt, randomly generated at the ‘root’ – i.e., the edge connecting the two oldest ancestors – from which 3 nt are randomly mutated at each internal node
• Segment 3: a ‘random’ part of 7 nt, randomly created for each training sequence independent from all other sequences in the training set

### 2.3 Interpreting phylogenetically informative features

Both siamese loss and quartet loss guide the neural network in reconstructing the given reference tree, thus yielding a phylogenetically informative latent space. In order to judge whether these features are biologically plausible, additional methods need to be applied to interpret neural networks and their output.

*In silico mutagenesis*  We used in silico mutagenesis analysis as one such explainability approach. Each nucleotide in the sequence was systematically mutated to any of the three other possible nucleotides, generating four sequences for each position, which were passed to the model. The resulting model output was normalized using the L1 norm, and a value of 0.25 was subtracted from each normalized prediction, formally obtained as

$$P_{{\text{norm}}_{i}}=\frac{P_{i}}{\sum_{j=1}^{4}|P_{j}|}-0.25.$$

The corrected normalization accounts for the baseline expectation of random predictions across four possible outcomes, allowing for a more meaningful comparison of the influence of each nucleotide mutation. The resulting values were visualized as a motif with logomaker [42].

*Relative nucleotide relevance for assessing latent spaces*  In order to assess the validity of the latent spaces inferred by the different models, a statistical test is needed to measure whether a given latent space variable is consistent with the tree topology. In our experiments, we tested this at the two subtrees branching at the root of the tree. Latent features that distinguish these two branches can be uniquely identified in a straightforward manner in all models that we trained. We henceforth refer to these features as *root features*.

For a model obtain during a certain epoch, we identify all such root features. For each root feature *f*, we used in-silico mutagenesis to obtain an attribution value for each sequence position *i* and each of the four nucleotide characters. After accumulating attributions across all four nucleotide characters and then normalizing across all sequence positions yields the *relative nucleotide relevance* $a_{i}^{f}$ at position *i*.

The basic idea behind this approach is that we expect the relevance in the evolving Segment 2 of the generated sequences to be significantly higher than in the random tail in Segment 3 if the root feature *f* is informative with respect to the underlying reference tree. To quantify how clearly the model localized relevance to the informative part of the sequence, we calculated for each root feature *f* the difference in average relevance between the evolving *Segment 2* spanning sequence position $S_{2}=\{9,\ldots ,28\}$ and the random tail in *Segment 3* panning sequence position $S_{3}=\{29,\ldots ,36\}$ of the generated sequences:

$$\Delta^{f}=\frac{1}{\left|S_{2}\right|}\sum_{i\in S_{2}}{\overset{ˆ}{a}}_{i}^{f}-\frac{1}{\left|S_{3}\right|}\sum_{i\in S_{3}}{\overset{ˆ}{a}}_{i}^{f}$$

We then compared the distributions of $\Delta^{f}$ across models trained on the *correct tree*—the tree used to simulate sequence evolution—and models trained on a randomized tree with a Mann-Whitney U rank test. A higher $\Delta^{f}$ indicates that the model more strongly focused attribution on the informative region of the input.

*Feature interpretation using DeepLIFT*  DeepLIFT (Deep Learning Important FeaTures), a backpropagation-based explainability technique, was applied to identify and interpret the phylogenetically informative features learned by the model [36]. DeepLIFT assigns attribution scores to the input components. By calculating attribution scores relative to a user-defined reference input, DeepLIFT offers insights into the factors that cause one sequence to activate a specific feature, while another sequence does not.

Feature vectors were extracted from successfully trained models that accurately captured the relationships between species. These feature vectors represent various biological traits encoded in the species data. The value assigned to the feature reflects the degree to which the trait is detected in the input.

A specific feature of interest was selected for further analysis from these feature vectors. Sequences lacking the feature of interest were chosen as reference sequences, and DeepLIFT was used to calculate attribution scores for each sequence that expresses the feature relative to these references. The attribution scores quantify the contribution of the feature to the prediction of the model. Finally, the attribution scores were averaged across all sequences exhibiting the feature, providing an assessment of the overall influence of this feature on the model's performance.

## 3 Results

### 3.1 Evaluation of the loss functions on 16S rRNA

We implemented both the siamese and the quartet loss function and used these to train models to reconstruct a given phylogenetic tree. We tested these loss functions on a reference tree of six alpha-proteobacteria and 7 gamma-proteobacteria (Fig. 4a). To rigorously evaluate the potential of our loss functions, data known to reliably produce a phylogenetic tree were selected, allowing us to focus on the performance of the loss functions without concern for the presence of phylogenetic signal. The V1 and V2 regions of the 16S rRNA in proteobacteria are well-established for containing strong phylogenetic signals. Since these variable regions are found within the first 350 nucleotides, we used this portion of the 16S rRNA sequence for training.

We trained 10 independent models with each loss function and calculated the quartet score after each epoch (Fig. 4b). The quartet loss network achieved its maximum quartet score in only a few epochs, and produced a splits diagram that reflects all edges of the reference tree (Fig. 4d). However, since the quartet loss can also be minimized if the model returns trivial solutions (e.g. only zeros), this led to model collapse after 30 epochs in most cases (Fig. 4c). The siamese loss prevents tree degeneration by avoiding the pursuit of minimal output, ensuring stability throughout the training process. However, the siamese model also requires more time to optimize, suggesting that it may struggle with sparse data. Additionally, it primarily focuses on matching the edge lengths of the reference tree, which can be problematic if the tree's edge lengths are unreliable, potentially leading to overemphasis on this feature.

To address these limitations, we introduced a third loss function, the quartet+siamese loss, combining the quartet loss with a regularization term inspired by the siamese loss. In training 10 models with this combined loss, we observed that the model was quickly optimized, with the quartet score reaching a value of 1 within the first training epochs.

To visualize and analyze the distance matrices generated by these models, NeighborNet representations were computed using the SplitsTree software package [8], [19] (Fig. 4d-i). While all three loss functions were able to recreate all edges of the reference tree, the combined loss created a distance matrix that induces an almost perfect splits diagram. The combined quartet+siamese loss function proved to be the most successful in reconstructing the reference tree from the 16S rRNA sequences, including all internal edges, while adding less extraneous area to the splits compared to using the loss functions independently.

### 3.2 Interpretation of learned phylogenetic features from simulated data

Examining the feature vectors generated by the model, we found that certain features were consistently associated with certain branches of the reference tree (Supplementary Figure 1). Remarkably, the dimensionality of the latent space is not fully exploited, leaving a certain fraction of the latent space features empty in the form of all-zero vectors. As illustrated further in Supplementary Figure 3, this occurs consistently across different dimensions of the latent space for all three loss functions under consideration.

This prompted further investigation into the sequence properties connected to these features and methods to make them accessible and interpretable. We explored two different explainability approaches that assign attribution scores to the input data. The first method is in silico mutagenesis, which uses mutated sequences as input to the model and compares the output to those of the original sequence. The resulting attribution scores quantify the influence of each nucleotide on the prediction, allowing identification of the most critical sequence positions that drive the model's decisions. The second approach is DeepLIFT, which calculates attribution scores by comparing the activation of each input feature to a predefined reference input, in order to estimate the contribution of each feature to the model's output. Since the feature of interest is present in some sequences but absent in others, the most suitable reference input is the set of sequences where the feature's value is zero in the feature vectors. Thus, these sequences were chosen as the reference for calculating the attribution scores with DeepLIFT.

**Fig. 5:**
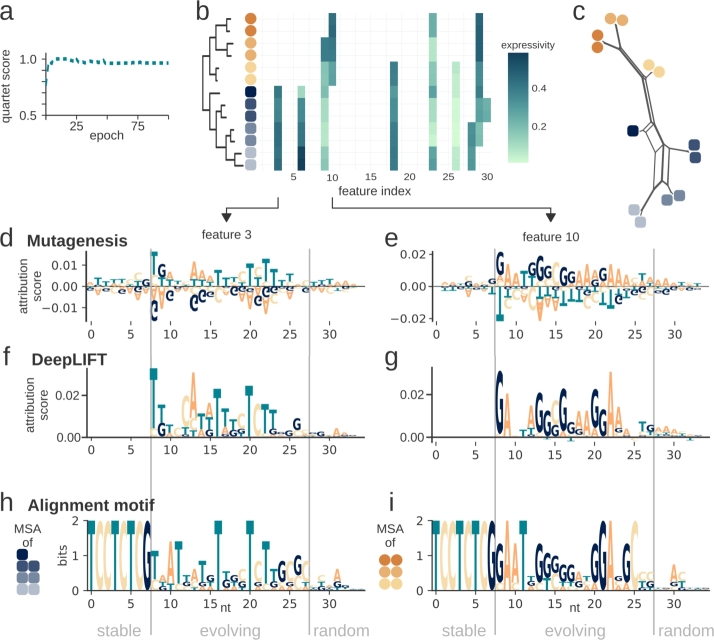
Extracting interpretable features of simulated sequences from a q+s model using DeepLIFT. The model was trained for 100 epochs (a). After 13 epochs, the feature vectors deriving from all 13 simulated sequences were extracted (b) and the Euclidean distances between the extracted feature vectors were reconstructed as NeighborNet (c). Features 3 and 10 were extracted from all feature vectors and used for in silico mutagenesis (d, e). The same features were extracted for a DeepLIFT analysis and the attribution score was averaged over all sequences that hold the feature, using all remaining sequences as reference (f, g). For comparison, the sequences holding the feature were also used to create a motif from the multiple sequence alignment (h, i).

To validate the methods for extracting phylogenetically informative features, we generated sequences with simulated evolutionary traits (Supplementary Figure 2). Each simulated sequence consists of three regions: a stable region that remains unchanged, a random region that is unique for each sequence, and a central evolving region which undergoes simulated evolution along a reference tree. Fig. 5d and e show the attribution scores from the simulated data for the features 3 and 10, calculated with in silico mutagenesis and averaged over the simulated sequences that contain the feature. The model focused on the evolving part of the sequence and almost ignored the stable and random parts. We also produced motifs from the same sequences, termed alignment motifs, with bits calculated according to the definition used for WebLogo [10] (Fig. 5h-i). The attribution scores of the in silico mutagenesis well reflect these alignment motifs. This applies both to positive scores for the species that carry the feature and to negative scores to those that lack it, while nucleotides that are present in both alignment motifs are assigned to small attribution scores (e.g. T at position 11).

Fig. 5f and g present the average attribution scores calculated using DeepLIFT for features 3 and 10 across all sequences that express the respective feature. Sequences lacking the feature were used as reference. The attribution scores generated by DeepLIFT align closely with those obtained from in silico mutagenesis. However, the differences in score magnitudes between the stable, evolving, and random regions of the input sequences are even more pronounced than the differences in the results generated with in silico mutagenesis. Unique motifs specific to the group of interest are clearly captured, while overlapping features are not detectable in the attribution scores, further emphasizing the distinctiveness of the unique regions.

In order to assess whether the models overfit the data, it is important to note that there is no straightforward way to cross-validate the models. Thus, in order to assess overfitting, we compared our models trained against the correct reference tree with models that were trained against a random tree that was obtained by random shuffling of the taxa. As displayed in Supplementary Figure 4, training on the randomized trees leads to only small and likely insignificant differences in the quartet score across the training process, indicating that a high quartet score is a necessary, but not sufficient criterion to judge the quality of the training process.

As a criterion that is not only necessary, but also sufficient, we have introduced a statistical hypothesis test that measures the gap in model-relevance between the evolving sequence positions on the one hand and the random sequence positions on the other hand, as described in Section 2.3. Our hypothesis is that this gap is significantly higher when training against the correct reference tree, compared to training against the randomized tree. The development of relevance of the different sequence parts can be seen in Supplementary Figure 5 for the correct reference tree and Supplementary Figure 6 for the randomized tree. These figures visually indicate that the relevance gap between the evolving sequence and the randomized sequence is significantly higher in the model obtained from the correct reference tree, compared to the randomized model. As displayed in Supplementary Figure 7, we conducted statistical significance tests across the training process. During the first epochs of the training, there is no significant difference between training against the correct tree vs. the randomized tree. After 10–15 epochs, the correct-tree model consistently gains significant relevance, which stabilizes after more than 20 epochs. Around epoch 80, significance drops, potentially indicating overfitting.

## 4 Discussion

In this study, we evaluated the effectiveness of two loss functions – siamese loss and quartet loss – in reconstructing phylogenetic trees using neural networks. Our experiments with 16S rRNA sequences revealed that while each loss function has distinct strengths and limitations, the best results were achieved by combining both losses with a regularization term. This combined approach capitalized on the advantages of each method, leading to more accurate and stable reconstructions of phylogenies.

The siamese loss function demonstrated robustness against model collapse by avoiding trivial minimization, which contributed to stable training throughout. However, its reliance on matching edge lengths from the reference tree introduces certain challenges. In cases where edge lengths are unreliable, the model may overemphasize this feature, making the siamese loss particularly sensitive to sparse data or datasets with less accurate phylogenetic information. Moreover, this approach is inherently limited to specific data types.

The quartet loss function generally converges within fewer epochs than the siamese loss functions. However, this does not translate to faster convergence in terms of computation time since the quartet loss function involves a larger number of terms, so that convergence time of training under quartet loss is similar to siamese loss. Quartet loss has the conceptual advantage that it does not require edge lengths in the reference tree, but utilizes only the tree topology, evading the question whether the phenotype evolves along the rates of evolution that match the reference tree. This makes it applicable to a wide range of input data, expanding to data types which do not naturally generate pairwise distance matrices, such as unalignable sequences or morphologies. When trained on 16S rRNA data, it quickly reached its optimal score. However, it was prone to model collapse after only a few epochs, as trivial solutions (such as returning all zeros) also minimize the loss. To counter this, we introduced a combined loss function (quartet+siamese) that stabilizes the training process by leveraging the strengths of both approaches. This combined loss function not only optimizes rapidly but also prevents model collapse, resulting in more robust phylogenetic reconstructions.

*Latent space interpretation*  In addition to evaluating the model performance through loss functions, we investigated the interpretability of the inferred latent space. In silico mutagenesis as well as DeepLIFT successfully highlighted specific regions of the sequence that contributed to model decisions, providing clear distinctions on the importance of stable, evolving, and random sequence regions.

A particular feature of our overall approach is that the interpretability of the latent space is crucial for validating the trained models in the lack of natural means of cross-validation. We have solved this by comparing the relevance of evolving sequences in relation to the relevance obtained from a random sequence tail. This can potentially guide the way of validation in future applications of the approach: Attaching a random tail can be easily accomplished for sequence data, but is also suitable for other data modalities such as vectorial *omics* data or image data, where random parts can be attached at fixed position. One can then use post-hoc interpretability methods to reason statistically whether attention is focused more on informative, signal-carrying parts than on random regions.

DeepLIFT focuses on unique motifs present in specific groups by subtracting the attribution scores of the reference sequences from those of the input sequences and effectively canceling out shared motifs. This makes the method particularly useful for identifying group-specific phylogenetic signals. However, DeepLIFT might also overlook inhibitory patterns when these patterns are present in both feature-positive and feature-negative sequences. In silico mutagenesis incurs higher computational costs, but also offers a more detailed analysis of which sequence patterns activate or inhibit specific features, providing a more comprehensive understanding of the underlying mechanisms. More generally, it may be promising to pursue non-convolutional neural networks such as transformer networks in combination with the tree-loss functions introduced in our manuscript. This will require the identification of suitable post-hoc interpretation methods to uncover learned motifs, and also involves some challenges to obtain feature representations with suitable distance measures.

*Limitations*  We have conducted our study on 16sRNA, which constitute a well-established phylogenetic marker gene that is expected to evolve along the species tree. However, beyond such well-established phylogenetic markers, this expectation must be treated with caution regarding how phenotypes may evolve. As recent studies have established in specific cases, eukaryotic phenotypes can be subject to incomplete lineage sorting [13], so that the phenotype tree is incongruent with the species tree. This must be taken into account when attempting to infer phylogenetically informative features, for example by careful selection of the reference tree. In such cases, it should be advantageous to select a gene tree of a gene involved in the phenotype under investigation instead of the species tree. In this context, inferring phylogenetically informative features may be a useful tool to obtain evidence how a particular phenotype has evolved.

Another limitation of our current study lies in the interpretability methods that we employed, which can only be used for alignable sequences. This problem is inherent to sequential data, and will differ in other use cases. In some instances, including sequence analysis, neural network architectures with better inherent interpretability such as transformer models may mitigate this problem. In general, interpretability methods are highly specific to the phenotype and the data obtained to investigate its evolution.

## 5 Conclusions

Looking ahead, the combination of the phylogenetic loss functions with attribution methods like DeepLIFT offers a promising framework for understanding phylogenetic relationships and the sequence features that inform them. The ability to extract and interpret phylogenetically informative features in a computationally efficient manner is crucial for future research, especially as datasets grow in size and complexity. Based on our findings, DeepLIFT or other backpropagation-based methods will likely become the preferred tools for extracting interpretable phylogenetic features from machine learning models. However, for comprehensive analyses requiring deep insights into both activating and inhibitory sequence patterns, in silico mutagenesis remains an invaluable tool, albeit computationally expensive.

In conclusion, this study demonstrates that training neural networks using phylogenetic loss functions combined with interpretability methods provides a viable tool to uncover functionally relevant features. The quartet and siamese loss functions, paired with DeepLIFT, represent a feasible approach that effectively captures and explains the phylogenetic signals in sequence data, paving the way for more refined and interpretable models in evolutionary biology.

## CRediT authorship contribution statement

**Vivian B. Brandenburg:** Writing – review & editing, Writing – original draft, Visualization, Validation, Software, Methodology, Investigation, Formal analysis, Data curation, Conceptualization. **Ben Luis Hack:** Writing – review & editing, Methodology. **Axel Mosig:** Writing – original draft, Supervision, Methodology, Conceptualization.

## Declaration of Competing Interest

The authors declare that they have no known competing financial interests or personal relationships that could have appeared to influence the work reported in this paper.

## Data Availability

The code for this project is available on GitHub (github.com/VivianBrandenburg/qtools) and Zenodo (version will be marked before publishing). Trained models from the shown examples are available on figshare (10.6084/m9.figshare.27263556).

## Appendix A Supplementary material

MMC Supplementary Figures 1–7. MMC
