# Supplementary material for: A quartet-based approach for inferring phylogenetically informative features from genomic and phenomic data
Source: Comput Struct Biotechnol J. 2025 Aug 22;27:3710–8. doi: 10.1016/j.csbj.2025.08.015 (PMC12398925; doi:10.1016/j.csbj.2025.08.015)
Supplement: MMC — Supplementary Figures 1–7. [file mmc1.pdf]

A quartet-based approach for inferring phylogenetically informative features from genomic and phenomic data
Supplementary Material

Vivian B. Brandenburg, Ben Luis Hack, Axel Mosig

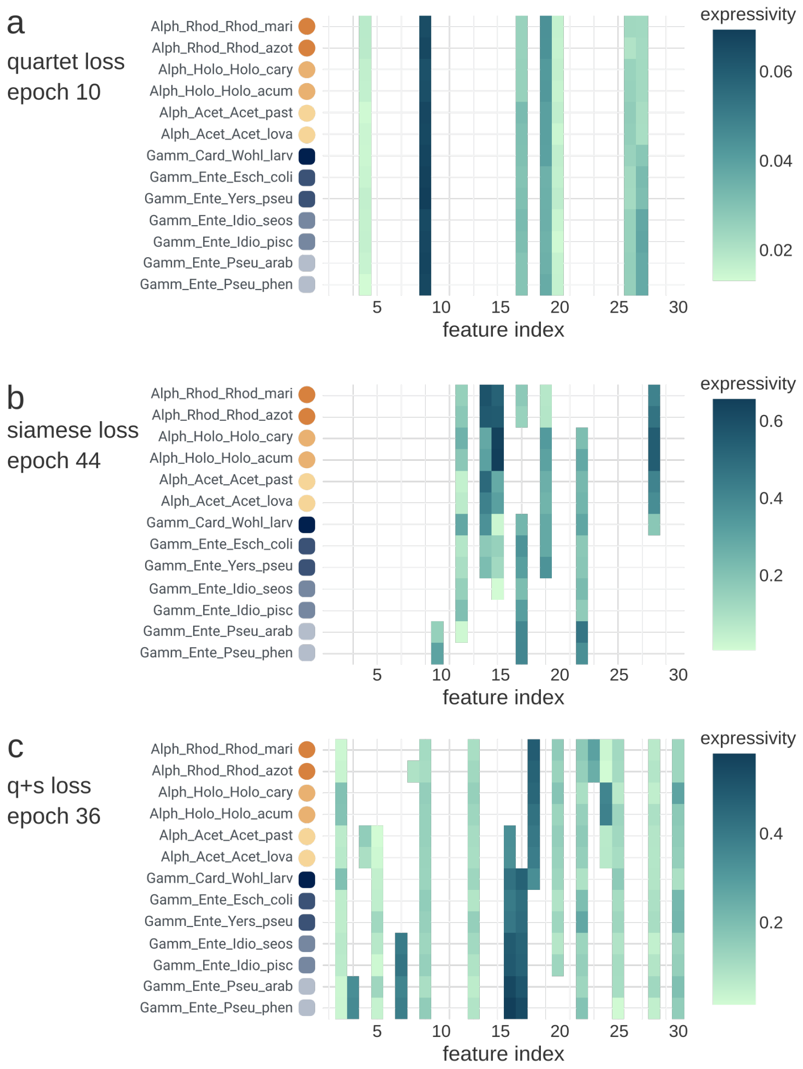

Supplementary Figure 1: Feature vectors produced from 16S rRNA data.

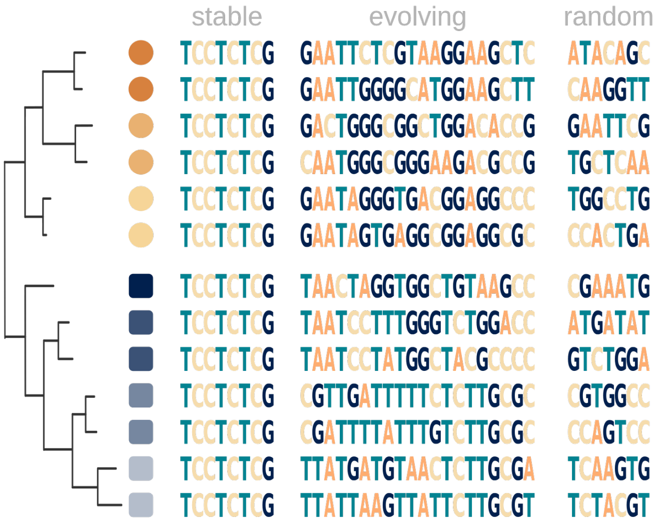

Supplementary Figure 2: Sequences of simulated data that were used for the extraction of interpretable phylogenetic features from trained models.

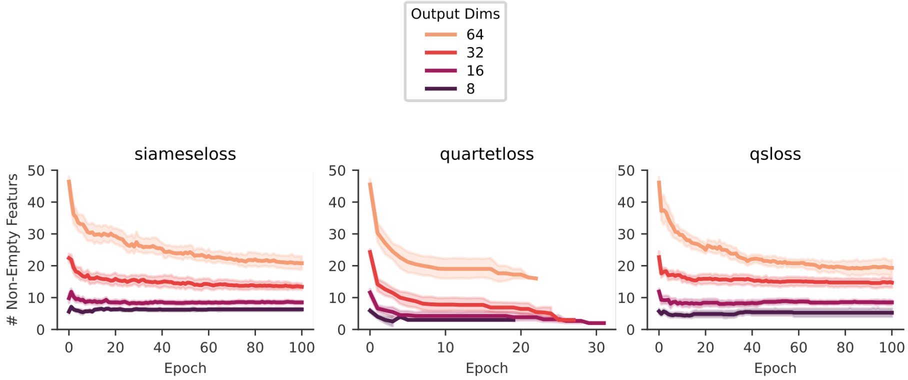

Supplementary Figure 3: Impact of output dimensionality on the prevalence of inactive features. The graphs display the average number of non-empty output features per epoch across 10 independently trained models for each dimensionality of the latent space. A feature is considered non-empty if it is active (i.e., assigned non-zero values) for at least one species within a given epoch. Shaded regions represent 95% confidence intervals. Higher output dimensionality is associated with an increased number of unused features, indicating greater sparsity and potential overparameterization.

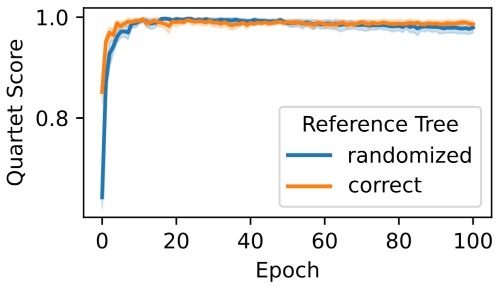

Supplementary Figure 4: Quartet score over training epochs for models trained on the correct reference tree (i.e., the tree that was used to simulate sequence evolution) and randomized reference tree. Mean quartet scores across 10 independently trained models are shown for each training epoch. Shaded regions represent 95% confidence intervals.

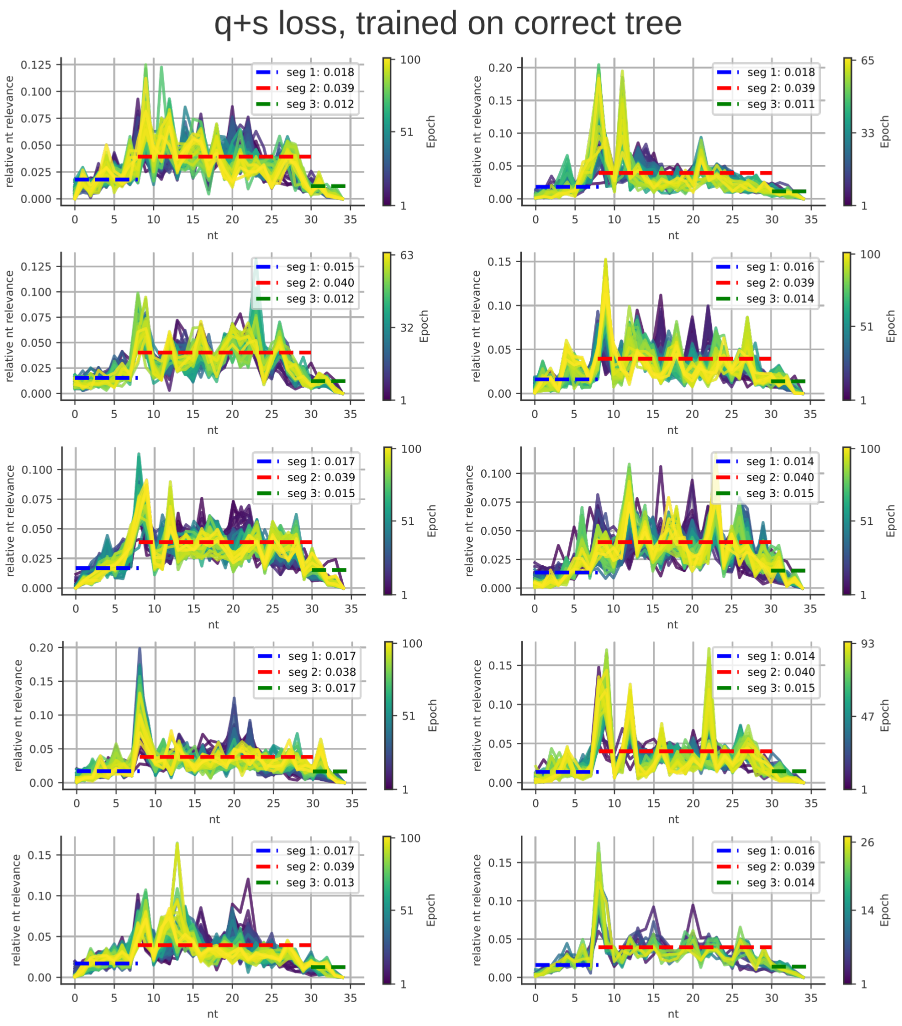

Supplementary Figure 5: Emergence of *relative nucleotide relevance* during training, *using the **correct** tree as reference tree.* Each plotted line represents the average normalized relevance profile for a specific epoch across all sequence positions. The relevance was derived from in silico mutagenesis by summing the absolute change in output across all nucleotide substitutions. Horizontal dashed lines indicate mean attribution levels for predefined sequence regions (seg. 1: stable, seg. 2: evolving, seg. 3: random, see Supplementary Figure 2).

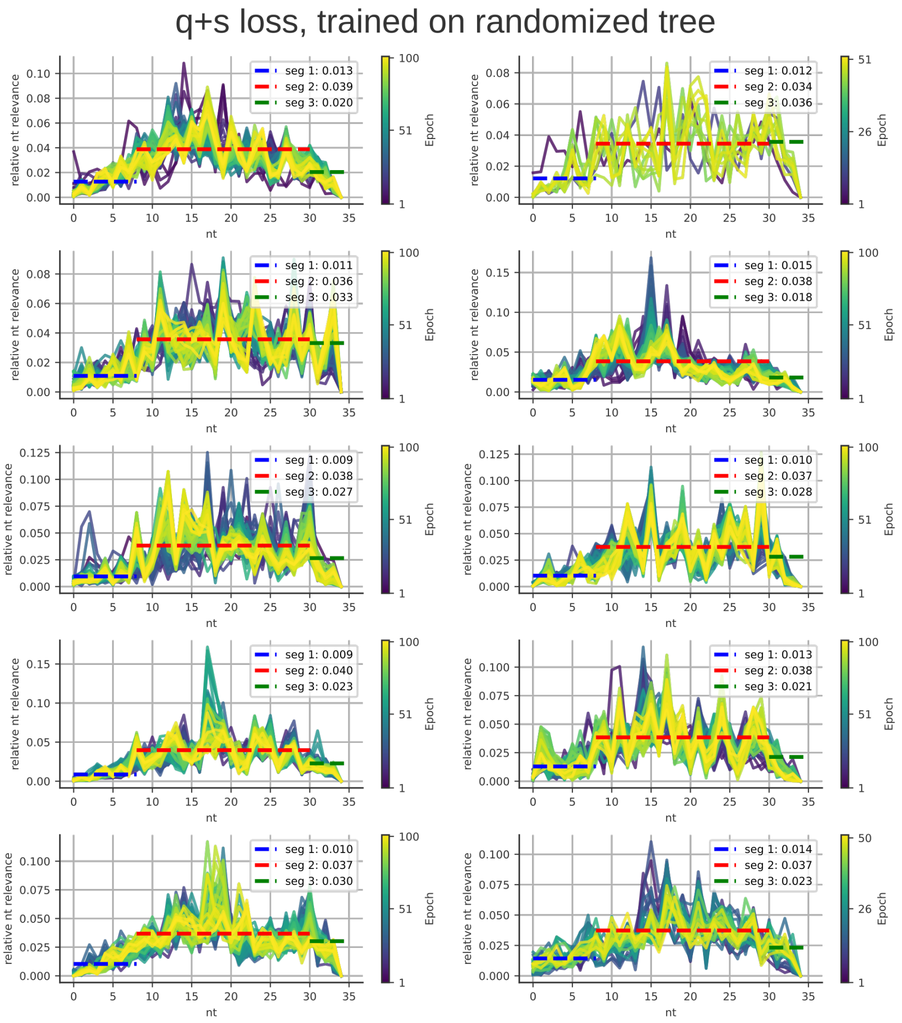

Supplementary Figure 6: Emergence of *relative nucleotide relevance* during training, *using the* ***randomized*** *tree as reference tree.* Each plotted line represents the average normalized relevance profile for a specific epoch across all sequence positions. The relevance was derived from in silico mutagenesis by summing the absolute change in output across all nucleotide substitutions. Horizontal dashed lines indicate mean attribution levels for predefined sequence regions (seg. 1: stable, seg. 2: evolving, seg. 3: random, see Supplementary Figure 2).

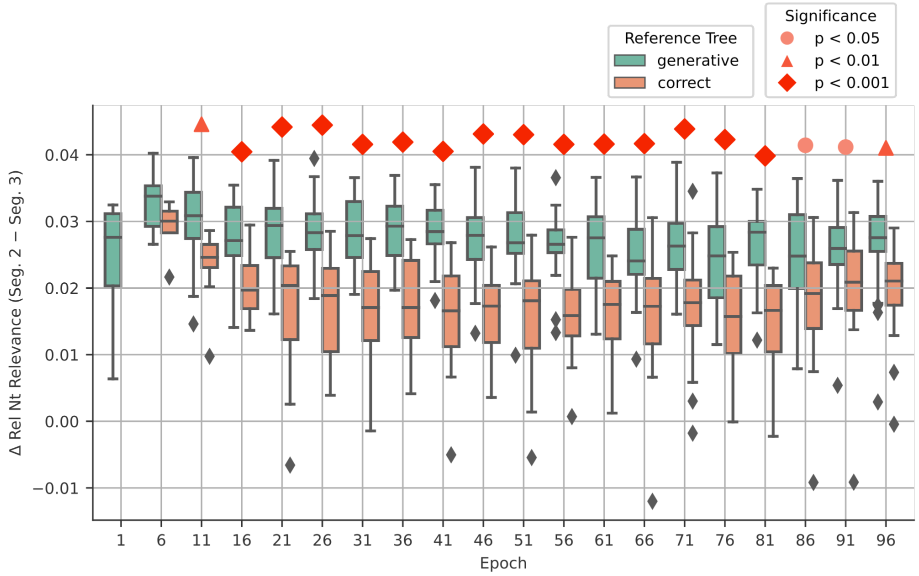

Supplementary Figure 7: Development of Δ *relative nucleotide relevance* of models trained on the correct vs. the randomized reference tree. Boxplots show the difference in average attribution between seg. 2 (evolving region) and seg. 3 (random region) for features selected across training epochs (measuring the gap between the red dashed line vs. green dashed lines, respectively, in Supplementary Figures 5 and 6, and testing the significance of the gap size between Supplementary Figures 5 and 6). The indicated p-values stem from a comparison between the correct-tree model and the randomized-tree model via Mann-Whitney U rank test. Intuitively speaking, we test whether the gap between the evolving region and the random region is significantly higher for the correct tree compared to the random tree. Models trained on the correct reference tree exhibit consistently higher Δ relevance, indicating stronger localization of attribution to phylogenetically informative positions. In contrast, models trained on a randomized tree display a weaker signal, supporting the effectiveness of the loss function in capturing the true phylogenetic structure. Remarkably, the Δ relevance does not differ significantly during the very first epochs of training, and consistently gains significance after 10–15 epochs. After more than 20 epochs, significance first stabilizes and then starts dropping around epoch 80, potentially indicating overfitting.
